# Efficacy of intravenous immunoglobulin in children with drug-resistant epilepsy

**DOI:** 10.3389/fneur.2026.1796553

**Published:** 2026-04-13

**Authors:** Aya Ebdalla, Morris H. Scantlebury, Juan P. Appendino, Alice Ho, Julia Jacobs

**Affiliations:** 1 Department of Pediatrics and Clinical Neurosciences, University of Calgary, Calgary, AB, Canada; 2 Alberta Children’s Hospital Research Institute, Calgary, AB, Canada; 3 Hotchkiss Brain Institute, Calgary, AB, Canada; 4 Division of Pediatric Neurology, Department of Pediatrics, University of Saskatchewan, Saskatoon, SK, Canada

**Keywords:** drug-resistant epilepsy, epilepsy, IVIg, pediatric epilepsy, seizure

## Abstract

**Purpose:**

Inflammation may contribute to drug-resistant epilepsy (DRE) in children and immunomodulatory therapies, including intravenous immunoglobulin (IVIG), have been described to reduce seizure burden. This retrospective study assesses the effect of IVIG on seizures in a tertiary epilepsy center.

**Methods:**

We performed a retrospective chart review of children with DRE using our Pediatric Epilepsy Outcome-Informatics database, which contains standardized information on 3,650 pediatric epilepsy patients collected at the point of care. Inclusion criteria were refractory seizures, regular follow-up and treatment with IVIG for at least 1 year. Patients with autoimmune encephalitis were excluded from the analysis. Sex, etiology, epilepsy syndromes, seizure types, antiseizure medications (ASM), adverse effects, and seizure frequency scores prior, 3 months post-, and 1-year post-IVIG were collected and statistically compared.

**Results:**

Sixty patients aged 2–18 years (mean ± SD, 11.8 ± 4.13) were included. Patients exhibited focal (38.3%, *n* = 23), generalized (36.7% (*n* = 22), or both seizure types (25.0%, *n* = 15). Overall, 36.7% (*n* = 22) demonstrated at least 50% seizure frequency reduction within 1 year of IVIG use, and 36.4% (*n* = 8) achieved seizure freedom. A significant reduction of seizures in individual patients was only observed in those with generalized seizures (*p* = 0.019). These findings could not be explained by changes in other ASMs.

**Conclusion:**

Long-term use of IVIG in pediatric patients can be an effective treatment for refractory seizures, even if exact mechanisms remain unclear.

## Introduction

Inflammation has long been suspected to play a role in the pathogenesis of drug-resistant epilepsy (DRE) and numerous clinical findings support the possible involvement of the immune system in epilepsy (1–3). The successful use of corticosteroids and IFN- *α* in certain epileptic syndromes provides early clinical support for immune involvement. In addition, increased cytokines in serum and cerebrospinal fluid following seizures, as well as increased expression of pro-inflammatory molecules in brain biopsies from DRE patients, further support the role of an inflammatory response and immune dysfunction in epilepsy (4–9). Recent work has further characterized the pro-inflammatory immune environment associated with epilepsy, including interactions between immune signaling pathways and neuronal excitability that may contribute to epileptogenesis (10). Such evidence has raised the prospect of alternative adjuvant therapies targeting inflammatory pathways or modulation of the immune system, such as intravenous immunoglobulin (IVIG) for patients who remain resistant to standard anti-seizure medications (ASM) and surgeries (6, 11, 40). In neurological disorders with a clear immunological basis such as Guillain-Barré Syndrome or anti-NMDA encephalitis, IVIG has shown promising results (12, 13, 40), however its efficacy is less clear in non-immunological epilepsies among pediatric populations due to a lack of clinical trials and a highly variable response rate attributable to heterogeneity in study designs, treatment modalities, and outcome parameters (Table 1) (6, 23–26, 40).

**Table 1:** Select pediatric studies investigating the use of IVIG for treating DRE.

Study group	Publication, study type	Patients (n)	Male/female	Mean age, (range, years)	Seizure types	IVIG frequency (weeks)	Outcome
Studies including participants with immune-mediated etiologies	Gross-Tsur et al. (14), prospective open-label study	9	3/6	5 (1.1–9.2)	Various	2–4	Complete remission in 44.4% of patients and seizure reduction between 25 and 50% in 33.3%.
	van Rijckevorsel-Harmant et al. (15), double-blind trial	61 (48 IVIG, 18 placebo)	42/19	23.3 (2–46.7)	Focal, generalized	0.15–3	Seizure reduction only observed in subgroup of patients with focal epilepsy.
	Billiau et al. (4), open-label prospective study	13	8/5	6.9 (1.6–25.8)	Various	3	Seizure reduction by at least 50% in 31% of patients and by 25% in 23%.
	Mikati et al. (16), open-label prospective study	37	23/14	9.94	Focal, generalized	4	Seizure reduction by at least 50% in 43.2% of patients and seizure freedom in 15%.
	Geva-Dayan et al. (17), retrospective study	64	35/29	3.2 (0–12)	Various	4–6	29.6% of patients responded to IVIG with either complete (*n* = 9) or partial resolution in (*n* = 10). West Syndrome patients were unresponsive to IVIG.
	Bello-Espinosa et al. (18), retrospective chart review	27	12/15	7.5 (3–17)	Various	3–12	Seizure reduction by 50% or more in 81.4% of patients.
	Al Amrani et al. (19), meta-analysis	8	5/3	9.75 (1–16)	Various	3–4	Seizure reduction by 50% in 75% of patients.
	González-Castillo et al. (20), retrospective case–control	38	26/12	6.12	Various	3	Seizure reduction by at least 50% in 52% of patients after 2 months and in 86% after 4 months.
Studies excluding participants with autoimmune etiologies	Tang-Wai et al. (21), retrospective chart review	51 (26 IVIG, 25 prednisone)	-	2.16 (2–13)	Focal, generalized	3–4	Mean seizure reduction by 65.4% in 84.6% of IVIG patients and by 75–100% in 24% of prednisone patients. Further, normalization of EEGs was observed in *n* = 3 (13.6%) and reduced spike frequencies in *n* = 5 (22.7%) of IVIG responders.
	Uran et al. (22), prospective open-label study	10	8/2	5.9 (4–8)	Focal, generalized, infantile spasms	1–4	Seizure reduction by 50% in 60% of patients and seizure freedom in 10%.

At our center at the Alberta Children’s Hospital, favorable effects of IVIG were previously observed in a retrospective study conducted by Bello-Espinosa et al. (18) wherein 22 of 27 pediatric DRE patients treated with IVIG demonstrated seizure frequency reduction by at least 50%, with the greatest reductions observed in patients with structural-metabolic (*n* = 3, 11.1%) and unknown aetiologies of epilepsy (*n* = 13, 48%). Given that up to 10% of new-onset childhood epilepsies may have an autoimmune basis with 70% of children with unknown etiology being antibody positive, it may be hypothesized that many responders in the previously reported study may in fact have an immune-mediated component to their seizures (18, 27). Therefore, investigating the efficacy of IVIG among children with genetic and structural aetiologies, in addition to those with unknown aetiologies, remains of paramount importance for identifying specific subgroups which may benefit most from IVIG treatment. Further, the limited number of studies investigating the relationship between IVIG efficacy and seizure types are conflicting. van Rijckevorsel-Harmant et al. (15), report the greatest reduction in seizure frequency among patients with partial seizures (now known as focal seizures), whereas Bedini et al. (28) and Mikati et al. (16) report a greater reduction of generalized seizures following IVIG treatment. On the contrary however, a number of studies report no relationship between seizure or epilepsy types and IVIG treatment outcome (17, 21).

Given the variability of results reported in the literature, the consensus for IVIG as an adjuvant therapy for treating children with DRE remains elusive (41–43, 45). In the present study, we report a retrospective analysis of a pediatric cohort with DRE treated with IVIG to improve the knowledge of the safety and efficacy of IVIG. We examined the effect of age, etiology, seizure types, and concurrent use of antiseizure medications on IVIG treatment outcomes. Adverse effects and reasons for terminating IVIG therapy were also evaluated.

## Methods

**Figure 1:**
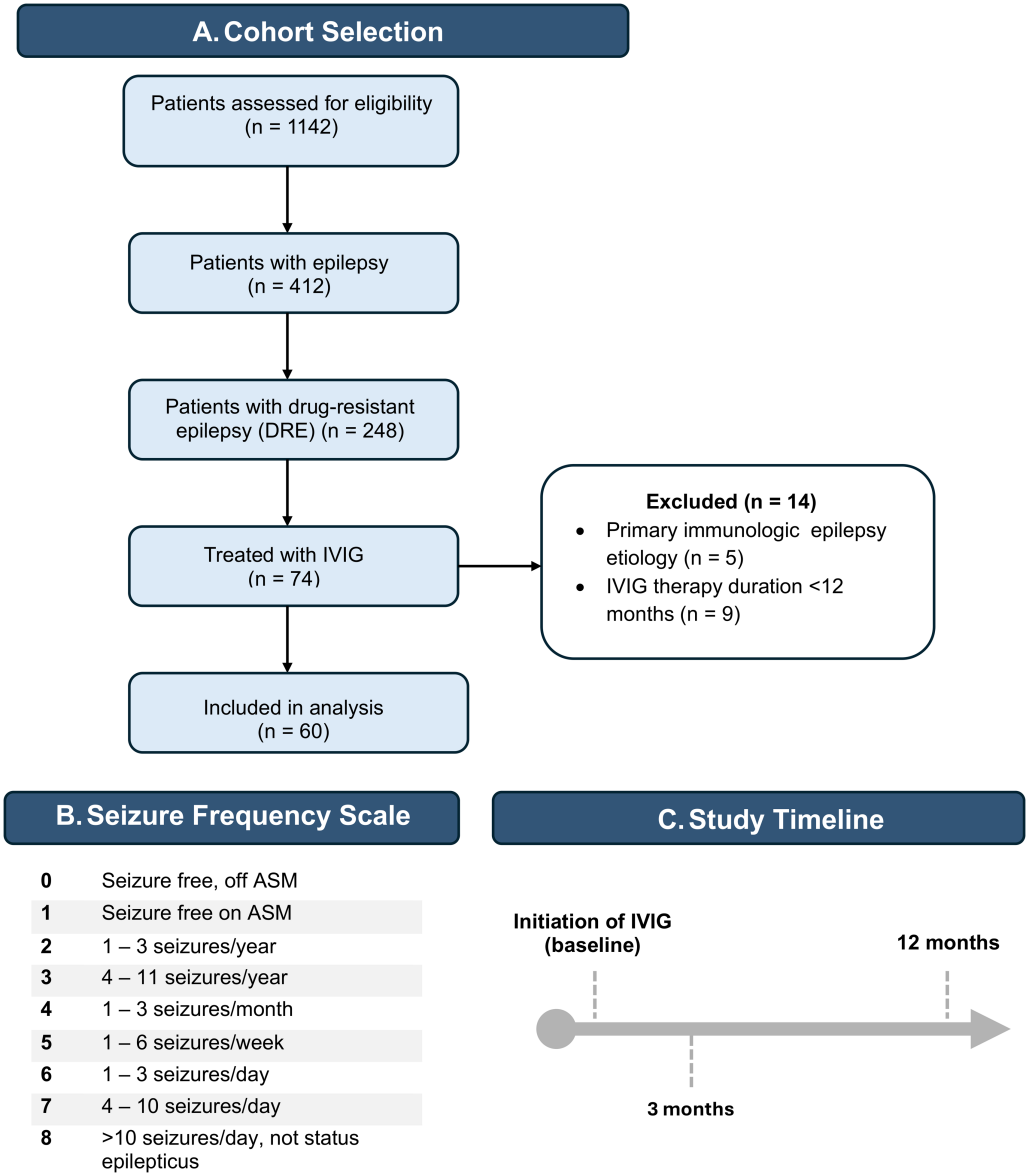
Study design and cohort selection. **(A)** Flow diagram showing cohort selection for DRE patients treated with IVIG. **(B)** Seizure frequency scale used for outcome classification. **(C)** Study timeline demonstrating data collection time points at baseline (IVIG initiation), 3 months post-IVIG, and 12 months post-IVIG follow up appointments.

We performed a retrospective chart review of children with DRE treated with IVIG for at least 1 year within neurology clinics at the Alberta Children’s Hospital. DRE was defined as per International League Against Epilepsy criteria: failure of adequate trials of two tolerated and appropriately chosen and used antiepileptic drug schedules to achieve sustained seizure freedom (29, 44). Patients were identified from our prospective Pediatric Epilepsy Outcome-Informatics database which contains standardized data on 3,850 + pediatric patients with epilepsy, collected at the point of care, displayed in Tableau Dashboards (Supplementary Figure S1) (30). The database may be filtered for various parameters, including medication. After selecting IVIG, 74 patients from our center with DRE who were not surgical candidates were identified. Children with epilepsy resulting from autoimmune epilepsy were excluded from the study (*n* = 5). The study design and patient selection process are summarized in a flow diagram (Figure 1A).

Basic demographic data included sex assigned at birth and age. Etiology, epilepsy syndromes, seizure types and ASM treatment history were extracted from the database. Main seizure types included focal-onset (including focal to secondary bilateral) and generalized-onset seizures (including atonic, tonic, myoclonic, absence and tonic–clonic). Etiology was categorized as genetic, structural, or unknown. Patients in the genetic and structural categories were assigned to respective categories based on results of previous genetic and neuroimaging studies, respectively. When the etiology remained elusive, patients were labeled as unknown and assigned to the unknown category. Epilepsy syndromes were also recorded. When assessing the effect of seizure type on IVIG treatment outcome, patients were seither grouped as having focal-onset, generalized-onset seizures, or both seizure types. This is a basic separation available in our database. ASM treatment history included the addition or removal of ASM with the initiation of IVIG therapy, and up to 1 year after IVIG therapy began. The number, type and dosages of ASM were recorded. IVIG dose and frequency was determined on a case-by-case basis by the treating neurologist. Doses ranged from 0.35 g/kg to 1.0 g/kg and frequency ranged from every 3 weeks to 12 weeks, although all patients initially start with a 4-week frequency for at least 3 months. The most common regimen was 0.4 g/kg every 4 weeks. Some patients had longer intervals, depending on tolerability. Adjustments to IVIG dose or frequency were determined by the treating neurologist, based on the seizure frequency scale (Figure 1B) (i.e., intervals shortened if seizures were sustained by the time the next monthly IVIG dose was due).

The documented treatment endpoint of our analysis was February 21 2021, and all patients received a minimum of 12 months of IVIG (4 treatments).

To quantify the efficacy of IVIG, we recorded seizure frequencies from three time points (Figure 1C): at the initiation of IVIG therapy, at least 3 months after IVIG therapy began (typically the first follow-up after changing a medication), and at 1 year after IVIG therapy. The minimum follow-up duration after the last dose modification was 6 months.

For each patient, an average seizure frequency score was assigned at each time point by using the patient-specific dashboards (Supplementary Figure S2) which provides seizure frequency scores for various seizure types across time.

Statistical analyses were performed using SPSS. The Repeated Measures ANOVA was used to detect statistical differences between independent variables (including etiology, seizure type and ASM use) and dependent variables (i.e., seizure frequency) across three time points. A *p* value of <0.05 was considered to indicate a statistically significant difference.

## Results

### Patient characteristics

Sixty-nine patients aged 2–18 years old (mean ± SD, 11.9 ± 4.13) treated with IVIG for non-autoimmune DRE met the inclusion criteria for our study. Nine patients (12.7 ± 4.33) discontinued IVIG prior to 1 year. The most common reasons for discontinuing therapy included sustainability issues (22.2%, *n* = 2) and no change in seizure frequency (22.2%, *n* = 2). No documented reasons were recorded for the remaining 5 patients that discontinued therapy. There was no statistically significant difference in age (*p* = 0.64), seizure type (*p* = 0.72) or etiology (*p* = 0.54) among the two cohorts. As such, all further analyses will focus on the cohort completing at least 1 year of IVIG therapy (*n* = 60). IVIg dosing varied between 0.35 g/kg to 1.0 g/kg. Most patients received 0.4 g/kg per dose. Minimum applied doses at last follow up were 0.35 g/kg/dose.

Among this cohort, 55.0% (*n* = 33) were male and 45.0% (*n* = 27) were female. No statistically significant differences between age and etiology across males and females were evident. The most common etiology was genetic (46.7%, *n* = 28), followed by structural (28.3%, *n* = 17) and unknown (25.0%, *n* = 15). Sixteen patients had a diagnosis of developmental epileptic encephalopathy with 10 having Lennox–Gastaut and 6 with Dravet syndrome. Within the sample, 38.3% (*n* = 23) patients exhibited focal seizures, 36.7% (*n* = 22) exhibited generalized seizure types, and 25.0% (*n* = 15) exhibited multiple seizure types. The types of generalized seizures exhibited within the cohort are outlined in Table 2. The most common generalized seizure type was tonic–clonic (33.3%), followed by myoclonic (26.7%) and atonic (15.0%). Focal seizures were more likely to be diagnosed in patients with structural etiologies, whereas patients exhibiting both focal and generalized seizures were more likely to have a genetic etiology (*p* < 0.001) (Table 3).

**Table 2:** Types of generalized seizures exhibited within DRE cohort treated with IVIG. Percentages exceed 100% as many patients experienced multiple seizure types.

Generalized seizure types	*N* (%)
Atonic	9 (15.0)
Tonic	11 (18.3)
Myoclonic	16 (26.7)
Absence	6 (10.0)
Tonic–clonic	20 (33.3)

**Table 3:** Demographic, seizure, etiology and ASM information.

Seizure type	*N*	Etiology	Sex	Average age	Number of ASMs prior to IVIG	Number of ASMs during IVIG
Focal	23	Structural = 52.2%* (*n* = 12) Unknow*n* = 24.7% (*n* = 5) Genetic = 26.1% (*n* = 6)	Male = 47.8% (*n* = 11) Female = 52.2% (*n* = 12)	12.3 ± 4.8	4.3 ± 2.1	4.5 ± 2.2
Generalized	22	Structural = 22.72% (*n* = 5) Unknow*n* = 40.9% (*n* = 8) Genetic = 40.9% (*n* = 9)	Male = 59.1% (*n* = 13) Female = 40.1% (*n* = 9)	12.7 ± 3.4	3.9 ± 1.7	5.0 ± 1.8
Various	15	Genetic = 73.3%* (*n* = 11) Unknow*n* = 26.7% (*n* = 4) Structural = 0%	Male = 60.0% (*n* = 9) Female = 40.0% (*n* = 6)	11.0 ± 4.5	5.4 ± 1.9	5.3 ± 2.0
Total	60	Structural = 28.3% (*n* = 17) Unknow*n* = 25.0% (*n* = 15) Genetic = 46.6% (*n* = 28)	M = 55.0% (*n* = 33) *F* = 45.0% (*n* = 27)			

*Significant at the *p* < 0.001 level.

All patients had been previously tried on at least 2 ASMs, and no significant difference was observed between the number of ASMs prior to IVIG among the focal (4.3 ± 2.1), generalized (3.9 ± 1.7) and various (5.4 ± 1.9) seizure subgroups. Additionally, no significant difference between number of ASMs across the groups was observed during IVIG therapy (Table 3).

### Overall effect of IVIG

Overall, 36.7% (*n* = 22) of patients exhibited a seizure frequency reduction by at least 50% within 1 year of IVIG use and 36.4% (*n* = 8) of these patients achieved seizure freedom (13.3% of the total population). Most patients experiencing a seizure reduction by 50% were diagnosed with genetic (54.5%, *n* = 12), followed by unknown (27.3%, *n* = 6), and structural (18.2%, *n* = 4) etiologies. 3.3% (*n* = 2) experienced a seizure frequency reduction less than 50 and 38.3% (*n* = 23) experienced no change in seizure frequency. Among patients achieving ≥50% seizure reduction (*n* = 22), the largest proportion had genetic etiologies (54.5%, *n* = 12), followed by unknown (27.3%, *n* = 6) and structural etiologies (18.2%, *n* = 4). However, a chi-square analysis showed no significant association between etiology and treatment response (*χ*^2^(2) = 1.80, *p* = 0.41) (Table 4).

**Table 4:** Seizure reduction by etiology.

Etiology	Total patients (*N*)	≥50% seizure reduction *N* (%)	<50% or no change *N* (%)
Genetic	28	12 (42.9)	16 (57.1)
Structural	17	4 (23.5)	13 (76.5)
Unknown	15	6 (40.0)	9 (60.0)
Total	60	22 (36.7)	38 (63.3)

### Clinical factors that may predict IVIG treatment outcome

#### Etiology

**Figure 2:**
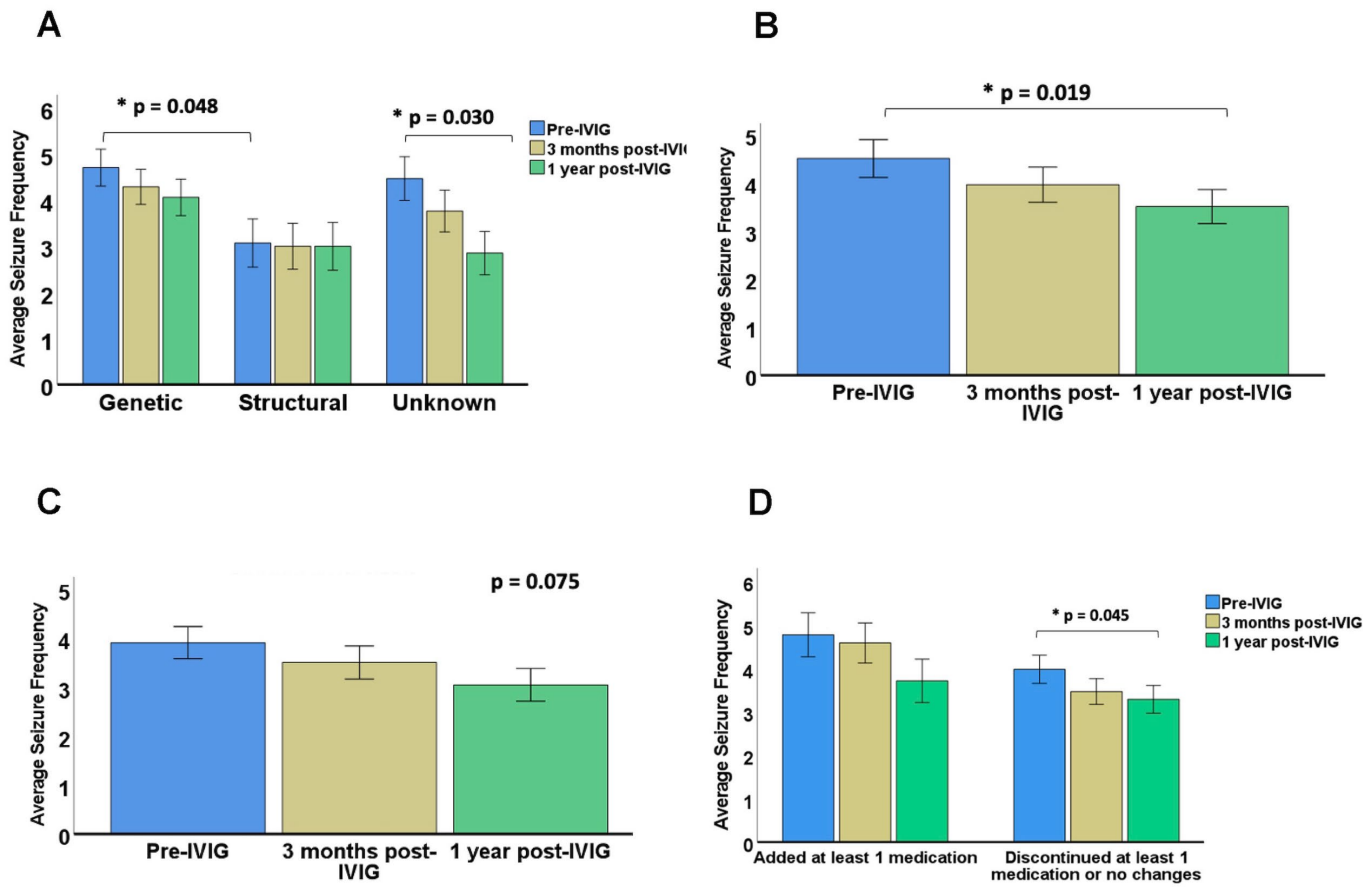
Changes in seizure frequency following IVIG therapy. **(A)** Mean seizure frequency by epilepsy etiology before IVIG therapy, 3 months post-IVIG therapy, and 1 year post-IVIG therapy. **(B)** Mean seizure frequency among patients with generalized seizures. **(C)** Mean seizure frequency among patients with focal seizures. **(D)** Mean seizure frequency stratified by ASM regimen changes. Error bars represent standard error of mean. *p*-values indicate comparison with baseline.

Patients with genetic etiologies exhibited higher baseline seizure frequency scores compared to children with structural etiologies (*p* = 0.048) (Figure 2A). A significant reduction in seizure frequency was observed among patients with unknown etiologies between baseline and 1 year time points (*p* = 0.03). However, no significant seizure frequency reductions were observed across time among the confirmed genetic etiology subgroups.

#### Seizure types

To analyze the relationship between seizure type and IVIG treatment outcome, we compared seizure frequencies across the 3 time points among patients with focal (*n* = 23), generalized (*n* = 22), or various seizure types (*n* = 15). After 1 year of IVIG therapy, there was a significant reduction in seizure frequencies among patients with generalized seizure types (*p* = 0.019) (Figure 2B). No significant reduction was demonstrated among patients exhibiting focal seizures (*p* = 0.075) (Figure 2C) or both seizure types (*p* = 0.095).

#### ASM use during IVIG therapy

To discern the effect of IVIG on seizure frequency reduction, we examined the effect of changes to ASM regimen during IVIG therapy. Medication changes could be assessed in 59 of 60 patients. A total of 16 (27.1%) patients required escalation of antiseizure medications during IVIG therapy, while 44 (74.6%) had stable or reduced ASM regimens. Specifically, 10 (17.0%) patients had no change in ASM regimen, 11 (18.6%) discontinued one ASM, and 22 (37.3%) discontinued two or more ASMs. A significant reduction in seizure frequency at the one-year time point compared to baseline was observed among the group with stable or reduced ASM regimens (*p* = 0.045) (Figure 2D). However, no significant reduction in seizure frequency was observed among patients requiring escalation of antiseizure medications during IVIG therapy.

#### Adverse effects

**Figure 3:**
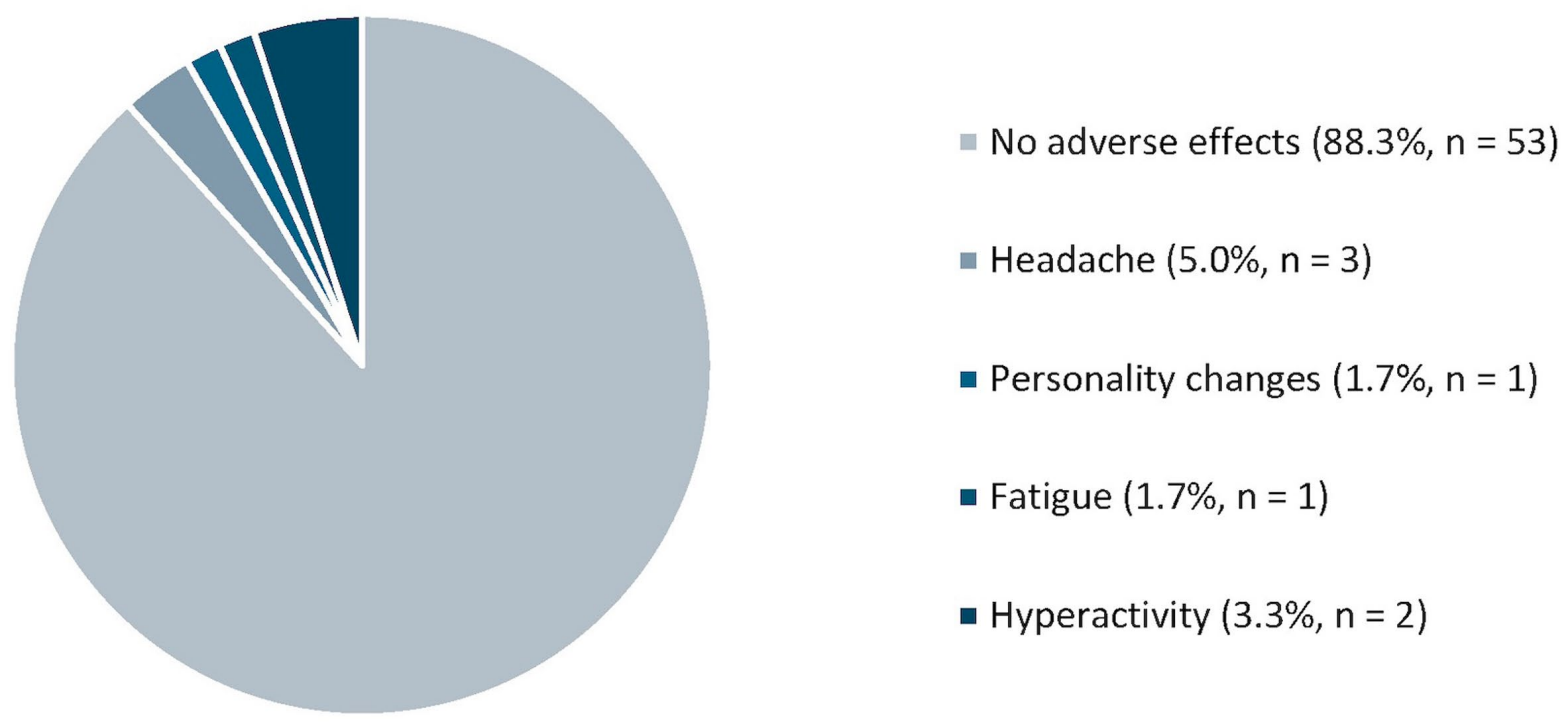
Reported adverse effects of IVIG therapy.

To assess the safety of IVIG, we examined adverse effects among patients using IVIG for at least 1 year (Figure 3). The majority reported no side effects (88.3%, *n* = 53) and no patients experienced greater than two side effects. However, 11.67% (*n* = 7) of patients reported side effects including headaches (42.86%, *n* = 3), hyperactivity (28.57%, *n* = 2), personality changes (14.29%, *n* = 1), and fatigue (14.29%, *n* = 1).

#### Reasons for discontinuing IVIG therapy

To assess the sustainability of IVIG, we examined reported reasons for discontinuing IVIG therapy after 1 year (Table 5). A total of 13 patients (21.7%) from the study cohort discontinued IVIG therapy with reported reasons including ineffectiveness (30.8%, *n* = 4), no resolution of EEG among CSWS patients (7.7%, *n* = 1) or other (23.1%, *n* = 3). For the remaining patients, no documented reasons were recorded (38.5%, *n* = 5).

**Table 5:** Reasons for discontinuing IVIG.

Duration of IVIG treatment	*N*	Ineffective	Sustainability	EEG did not resolve	Other	No reason documented
< 1 year	9	22.2% (*n* = 2)	22.2% (*n* = 2)	0%	0%	55.6% (*n* = 5)
> 1 year	13	30.8% (*n* = 4)	0%	7.7% (*n* = 1)	23.1% (*n* = 3)	38.5% (*n* = 5)

## Discussion

The present study confirms the potential value of IVIG for reducing seizure frequency among patients with DRE. Based on our findings, patients with generalized seizures were more likely to experience a reduction in their seizures after 1 year of adjuvant IVIG therapy, though no significant benefit was seen at 3 months of therapy (Figure 2B). IVIG was also beneficial in cases where the etiology of epilepsy remained unknown. Importantly, treatment was well tolerated, with 88.3% of our cohort reporting no adverse effects and the remaining 12% reporting mild, transient symptoms.

Except of the above mentioned patients with a syndrome diagnosis, all remaining patients had refractory epilepsy without a defined syndrome. This distribution provides meaningful context for interpreting our findings, particularly as prior studies, such as those by Mikati et al. (16) included different syndromic profiles, including Landau–Kleffner syndrome and infantile spasms in addition to Lennox–Gastaut syndrome. While some of these syndromes, including Lennox–Gastaut and Dravet, may exhibit immune alterations (31, 32), these are secondary phenomena rather than primary etiologies. In our study, patients with primary autoimmune etiologies of epilepsy, such as autoimmune encephalitis, were excluded from the study, to ensure the cohort represents predominantly non-autoimmune DRE. Thus, there is partial but not complete overlap between our cohort and previously studied populations, which likely contributes to variability in reported response rates across the literature.

The response rate of IVIG in DRE in the pediatric population is highly variable (41–43, 45). In prospective studies, IVIG was shown to reduce seizure frequency by at least 50% in 31% of patients (*n* = 7) (4) and in 43.2% of patients (16). In retrospective studies, the response rates were higher: 50% reduction or more in 52.0% of patients (*n* = 19) (20), 81.4% of patients (*n* = 22) (18), 84.6% of patients (21) and complete resolution in 47.37% (17). We report a seizure reduction of 50% or more in 36.7% of patients (*n* = 22) and seizure freedom in 13.3% of our sample, which constitutes 36.4% (*n* = 8) of patients exhibiting a seizure reduction by at least 50%. The time of peak effect was also variable among studies. In the study reported by Geva-Dayan et al. (17), the peak effect was achieved immediately or after 5 months of IVIG therapy, whereas Tang-Wai et al. (21) report a mean time of best seizure reduction at 9.8 weeks after the first infusion of IVIG. In our study, no effect was observed after 3 months of IVIG, however seizure frequency reduction was significantly reduced after 1 year of IVIG. Further studies including data collection at more frequent time points are needed to ascertain the peak time to effect of IVIG.

Although our data are encouraging, our study is not without limitations inherent to retrospective designs such as the lack of randomization, absence of control group, and limited control over certain variables. Many studies examining the efficacy of IVIG rely on parent accounts to quantify seizure reduction, which inevitably introduces recall bias. In our study however, we use a standardized seizure frequency scale to mitigate recall bias and account for the lack of control over variables in retrospective studies. Another potential limitation in our study is the spontaneous changes or improvement that occurs in epilepsy, which is frequently observed in self-limited epilepsy with centrotemporal spikes (seLECTs) and childhood occipital visual epilepsy (33), both of which were not present within our cohort.

Despite their challenges, observational studies remain an important source of information in cases where clinical trials are difficult to execute and provide a foundation for prospective studies. Our study contains one of the largest samples in pediatric epilepsy analyzing the effect of IVIG on seizure types and etiology in DRE patients, in addition to seizure frequency changes, while taking medication changes during IVIG therapy into consideration.

### Effect of etiology on IVIG treatment outcome

In our study, children with unknown aetiologies showed significant seizure reduction after 1 year, whereas those with genetic or structural epilepsies did not (Figure 2A). Interestingly, previous studies report mixed results. Geva-Dayan et al. (17) report a correlation between treatment response and etiology among subgroups of patients with epileptic encephalopathies, specifically in idiopathic but not symptomatic West Syndrome, and in Electrical Status Epilepticus in Sleep. However, no association between responsiveness and etiology was observed among children in their cohort with non-encephalopathic DRE. In another study, etiology was not found to be a predictor to IVIG responsiveness (16). Tang-Wai et al. (21) also showed no correlation between epilepsy etiology, type or duration and response. Interestingly, in the study conducted by Bello-Espinosa et al. (18), patients with >90% seizure reduction (*n* = 9) included both structural and unknown etiologies, although much of the subgroup had unknown etiologies for their epilepsies (62.5%, *n* = 5). However, analyses to ascertain the relationship between etiology and IVIG responsiveness were not completed.

In keeping with our findings, higher response rates in children with cryptogenic epilepsies, currently known as suspected genetic-metabolic etiology, compared to children with symptomatic epilepsies have previously been reported (34–36, 46). The culmination of these findings may be explained by the hypothesis that up to 10% of patients with unknown etiologies of epilepsy may indeed have an autoimmune basis (37, 38). Therefore, IVIG therapy may be less effective in patients with structural or monogenetic etiologies as the pathophysiology of such epilepsies likely involves less immune activation.

### Effect of seizure type on IVIG treatment outcome

#### Generalized seizures

In our study, we demonstrated a significant association between seizure type and IVIG responsiveness whereby patients with generalized seizures were more likely to respond to IVIG after 1 year, as compared to patients with focal seizures only (Figure 2C). In keeping with our findings, one study reported a greater seizure frequency reduction among patients with generalized seizures (16). Similarly, Billiau et al. (4) report a seizure frequency reduction among a cohort of patients primarily exhibiting generalized seizures (84.62% exhibiting various types of generalized seizures, 15.38% exhibiting focal seizures). Given that 40.9% of patients in our cohort with generalized seizures had an unknown etiology of epilepsy, it is unsurprising that a greater seizure frequency reduction was observed with generalized seizures since an autoimmune basis is hypothesized to underlie a certain proportion of patients with unknown etiologies (27, 38).

#### Focal seizures

Based on our findings, IVIG did not effectively reduce seizure frequency among the focal seizure subgroup. Although our findings within the focal seizure subgroup did not reach significance, previous studies do report a seizure frequency reduction in a subgroup of patients with partial epilepsy, which remained significant among patients with secondary bilateral seizures (15). Nonetheless, the data concerning the efficacy of IVIG for specific seizure types remains equivocal (16).

### Effect of changes in other ASMs on IVIG treatment outcome

On average, patients included in our study were using 4.43 ± 1.96 ASMs prior to beginning IVIG therapy. Given the refractoriness of our cohort, medication changes were often made by the prescribing neurologist at follow-up appointments however, there was no significant difference in the number of ASMs during IVIG therapy (4.88 ± 1.99). In keeping with previous studies, this finding highlights the refractoriness of our cohort. In the study completed by Tang-Wai et al. (21), participants tried between 2–9 ASMs prior to IVIG, however the number of ASMs during IVIG therapy was not reported. Similarly, Bello-Espinosa et al. (18) reports a range of 2–10 ASMs tried before IVIG therapy with no change to the number of ASMs during IVIG therapy. Without considering medication changes during IVIG therapy, the efficacy of IVIG will be difficult to ascertain (6, 16, 39). As such, we attempted to mitigate this confound by comparing seizure frequencies between patients with and without ASM changes. In our study, seizure frequency reduction remained significant among patients who had no ASM changes or who discontinued an ASM during IVIG therapy (*p* = 0.045, Figure 2D). This finding is somewhat unexpected, as discontinuation of antiseizure medications would typically be anticipated to worsen seizure control. One possible explanation is that clinical improvement associated with IVIG allowed for medication reduction in some patients. While this observation may indirectly support a therapeutic effect of IVIG, causality cannot be established in this retrospective cohort.6.

## Conclusion

Studies support the potential value of IVIG in DRE despite the mechanism of IVIG in DRE remaining unclear. In the current study, we confirm the possible efficacy of IVIG in refractory epilepsy with a seizure frequency reduction by at least 50% in 36.7% of participants, primarily among participants with genetic and unknown etiologies. A third of these patients (36.4%) also achieved seizure freedom. Based on our data, generalized seizures were the most responsive to IVIG therapy. Our data also support the safety of IVIG, with 88% indicating no adverse effects.

In summary, IVIG therapy is a safe adjuvant therapy and should be considered early in children with refractory epilepsy, particularly among patients with genetic or unknown etiologies and among patients exhibiting generalized seizures. Further studies are needed to support this hypothesis however, and to delineate the peak time to effect, the ideal dose per epilepsy syndrome, and responsiveness to IVIG based on seizure type.

## Data Availability

The original contributions presented in the study are included in the article/Supplementary Material, further inquiries can be directed to the corresponding author.
