# Supplementary material for: Efficacy of intravenous immunoglobulin in children with drug-resistant epilepsy
Source: Front Neurol. 2026 Apr 13;17:1796553. doi: 10.3389/fneur.2026.1796553 (PMC13127252; doi:10.3389/fneur.2026.1796553)
Supplement: Supplementary file 1 [file Image_1.pdf]

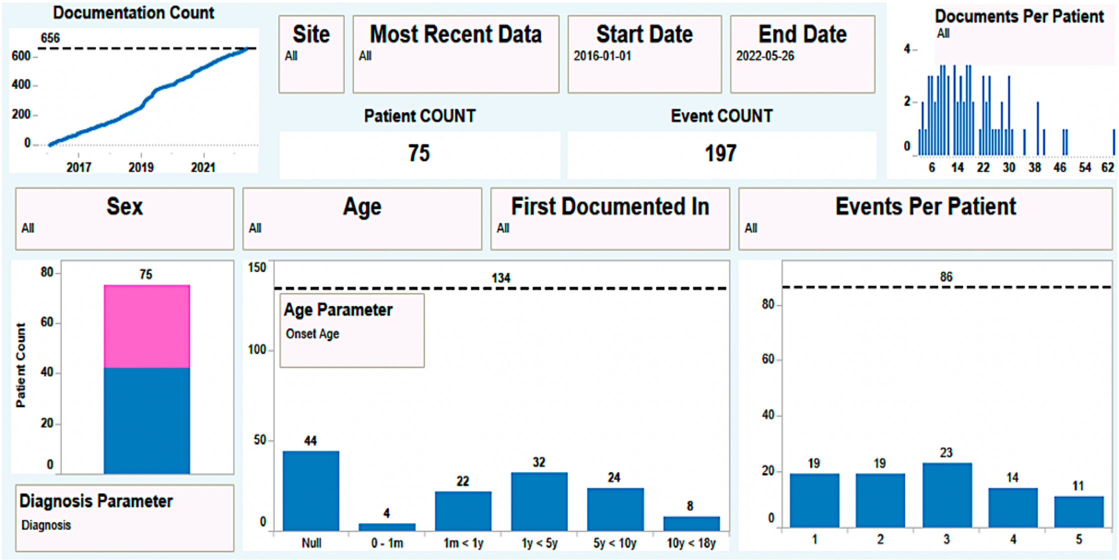

Figure S1. Tableau Dashboard displaying select data for DRE when the IVIG medication filter is applied. As of May 26, 2022, there were a total of 75 patients treated with IVIG, however at the time of the data collection, there were 74.
