# Supplementary material for: Efficacy of intravenous immunoglobulin in children with drug-resistant epilepsy
Source: Front Neurol. 2026 Apr 13;17:1796553. doi: 10.3389/fneur.2026.1796553 (PMC13127252; doi:10.3389/fneur.2026.1796553)
Supplement: Supplementary file 2 [file Image_2.pdf]

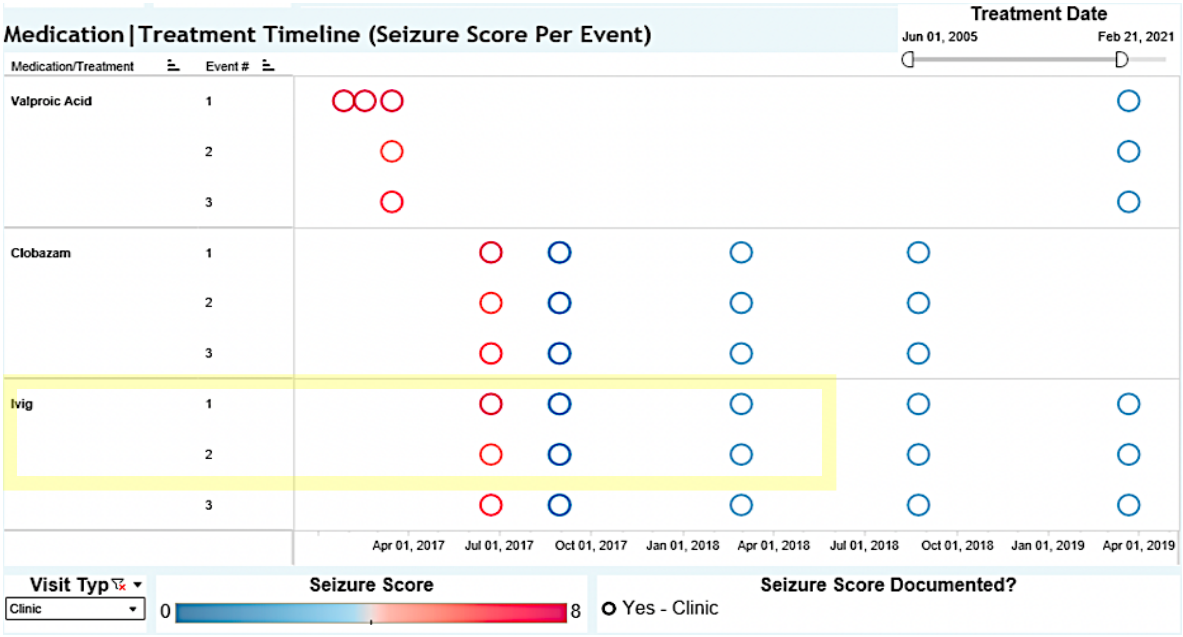

Figure S2. Treatment timeline obtained from Tableau Dashboards for a patient in the present study with response to IVIG therapy. Event number refers to number of seizure types. This patient had 3 seizure types. Red data points indicate higher seizure frequency scores, and blue data points indicate lower scores.
